# Feasibility of outpatient daycase local anaesthestic Rezūm™ without sedation

**DOI:** 10.1186/s12894-024-01471-2

**Published:** 2024-04-04

**Authors:** S Khadhouri, S Guillaumier, L Drummond, B Dreyer, C Clelland, F Al Jaafari

**Affiliations:** 1 NHS Fife, Kirkcaldy, Scotland, UK; 2 University of St Andrews, St Andrews, Scotland, UK

**Keywords:** Rezum, Local anaesthetic, Outpatient, Daycase, Bladder outflow obstruction surgery, Minimally invasive surgical treatment, BOO, BOO surgery, MIST

## Abstract

**Background:**

Rezūm™ is a relatively new bladder outflow obstruction (BOO) procedure that uses thermal energy through water vapour to cause necrosis of prostatic tissue. The standard delivery of this treatment is in an operating theatre under a general or spinal anaesthetic, or under local anaesthetic with sedation that requires patient monitoring.

**Methods:**

We propose an outpatient daycase method of delivering Rezūm™ under local anaesthetic without sedation, using a prostatic local anaesthetic block and cold local anaesthetic gel instillation into the urethra.

**Results:**

Preliminary results of our first thirteen patients demonstrate the feasibility of this new technique, with a mean pain score of 2.1 out of 10 on a visual analogue scale, a successful trial without catheter in all 13 patients (one patient voided successfully on second trial), a reduction in mean International Prostate Symptom Score (IPSS) from 20.6 to 5.4, and improvement in maximum flow from 8.8 ml/s to 14.4 ml/s. The complications were minor (Clavien-Dindo less than III) and included a UTI, minor bleeding not requiring admission, and retrograde ejaculation.

**Conclusions:**

We demonstrate that an outpatient local anaesthetic daycase service without sedation is feasible. This can be delivered in a clinic setting, reduce waiting times for BOO surgery, and increase availability of operating theatre for other general anaesthetic urological procedures.

## Background

Rezūm™ is a relatively new minimally invasive treatment for benign prostatic enlargement (BPE) to improve bladder outflow obstruction (BOO) [1]. Thermal energy through water vapour (steam) is delivered transurethrally into the prostatic lobes causing necrosis of tissue, which sloughs off with time thereby un-obstructing the prostatic urethra. The National Institute for Health and Care Excellence recommends it should be considered as a treatment option for people with moderate to severe lower urinary tract symptoms (LUTS) and a moderately enlarged prostate typically between 30 cc to 80cc [2]. This is based on evidence showing a significant improvement in symptom relief and improved quality of life that was durable for over 4 years [3]. Most centres in the UK deliver this service under general anaesthetic (GA), or under local anaesthetic (LA) with IV sedation [4]. One method using a Schelin catheter to deliver local intraprostatic anaesthesia transurethrally has been described and shows promising results for a Rezūm™ outpatient service [5]. A different method using methoxyflurane inhaler has been shown as a potential for use in day case Rezūm™, although this was used alongside oral lorazepam, oxycodone and simple analgesia [6]. We believe we are also one of the first urological units to deliver this treatment under LA alone via a different technique, but without sedation or oral analgesia, in an outpatient setting.

## Methods

### Infrastructure

Before commencing the delivery of an outpatient service for minimally invasive BOO procedures, the appropriate supporting staff and resources are required. This includes an admission bay where the patient can be consented and changed into a theatre gown. Staff should include at least a trained nurse, preferably a clinical nurse specialist (CNS) in BPE, and a supporting healthcare assistant (HCA). The same admission bay can be used for post procedure observations and recovery before discharge. A dedicated Consultant-led weekly clinic has been set up to deliver this service in our unit.

### Room setup

**Fig. 1:**
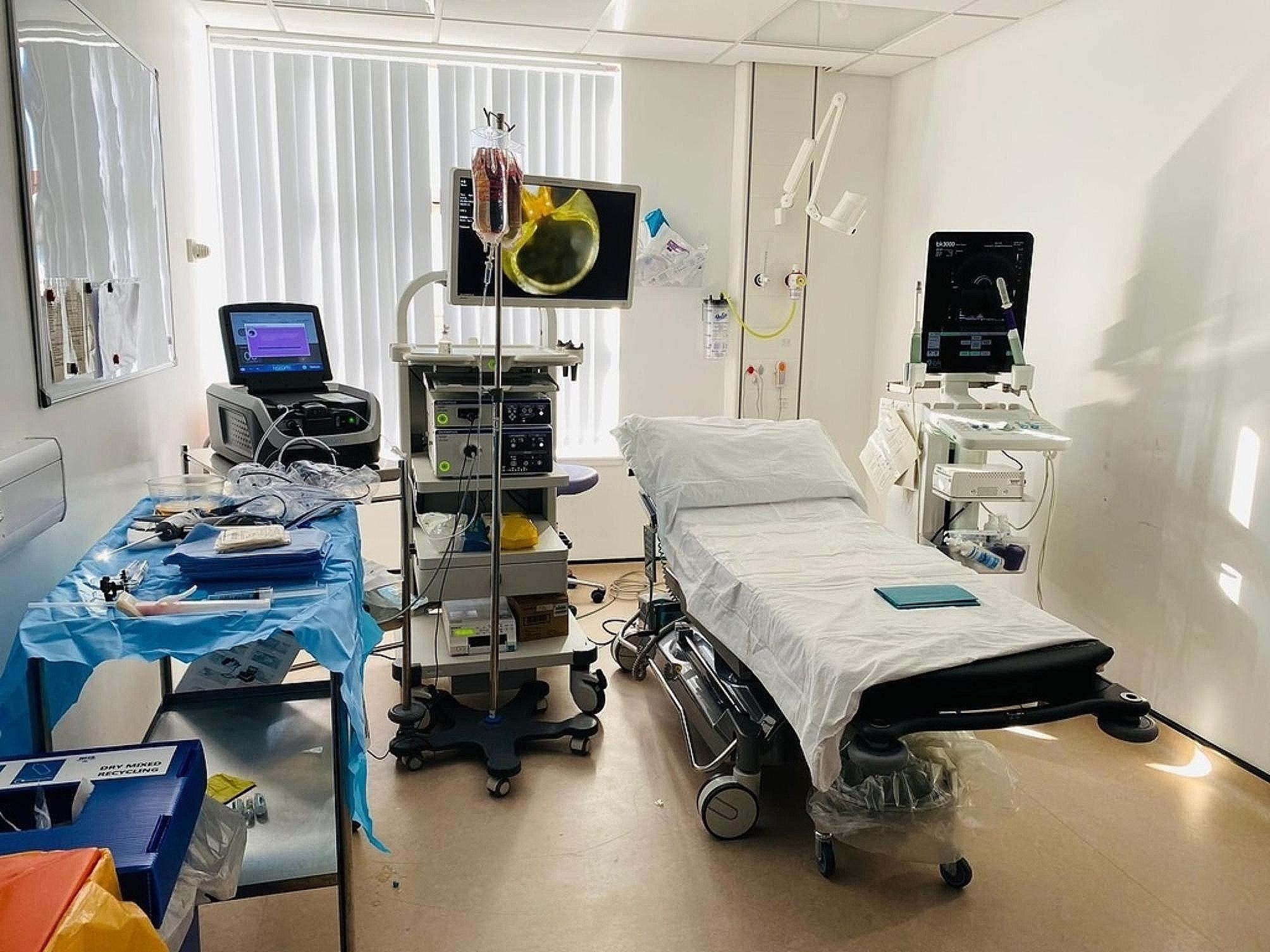
Room setup with ultrasound machine to the right of the couch, and cystoscopy stack, IV drip stand, Rezūm™ machine and scrub trolley to the left

The ultrasound machine (BK3000) is placed on the right side of the patient couch, and the cystoscopy stack and Rezūm™ machine on the left side at the head of the couch (Fig. 1). The scrub trolley is situated on the left of the surgeon’s chair at the foot of the couch.

**Fig. 2:**
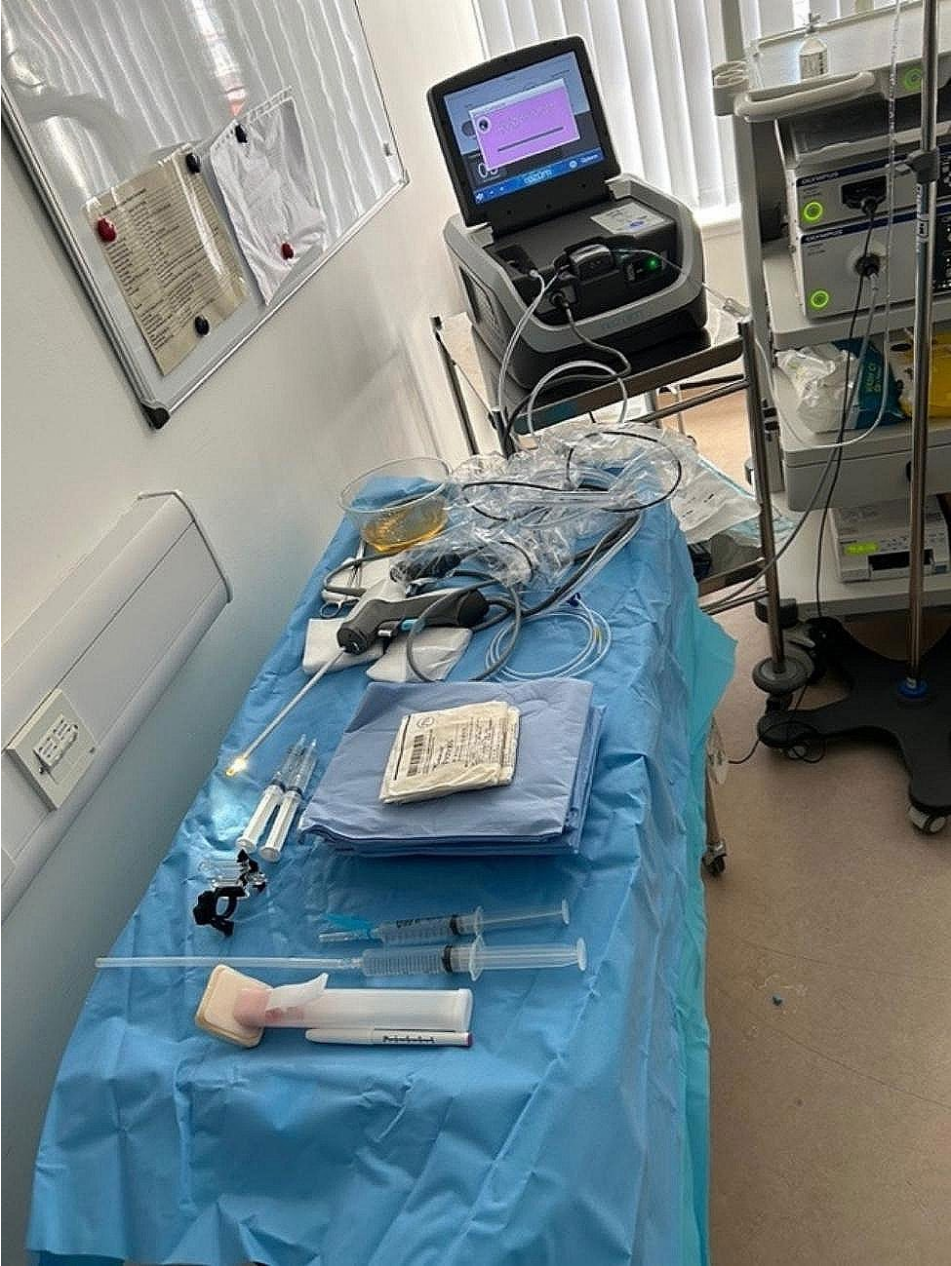
Scrub trolley equipment setup

### Equipment setup

The scrub trolley includes (Fig. 2):

- Chloraprep 3 ml applicator.
- Antiseptic cleaning solution (0.015% chlorhexidine gluconate + 0.15% Centrimide).
- Rampleys and gauze.
- Under buttock drape, leg drapes and abdominal drape.
- Two 11 ml syringes of local anaesthetic gel.
- 10 ml 1% lidocaine with adrenaline 1:200,000 attached to a blue hypodermic needle.
- 20 ml 1% lidocaine without adrenaline attached to a spinal needle (20G).
- PrecisionPoint^™^ Transperineal access system with 15G access needle.
- Marker pen.
- Rezūm^™^ device.
- 30-degree cystoscope and camera and light lead in sterile cover.

### Ultrasound setup

**Fig. 3:**
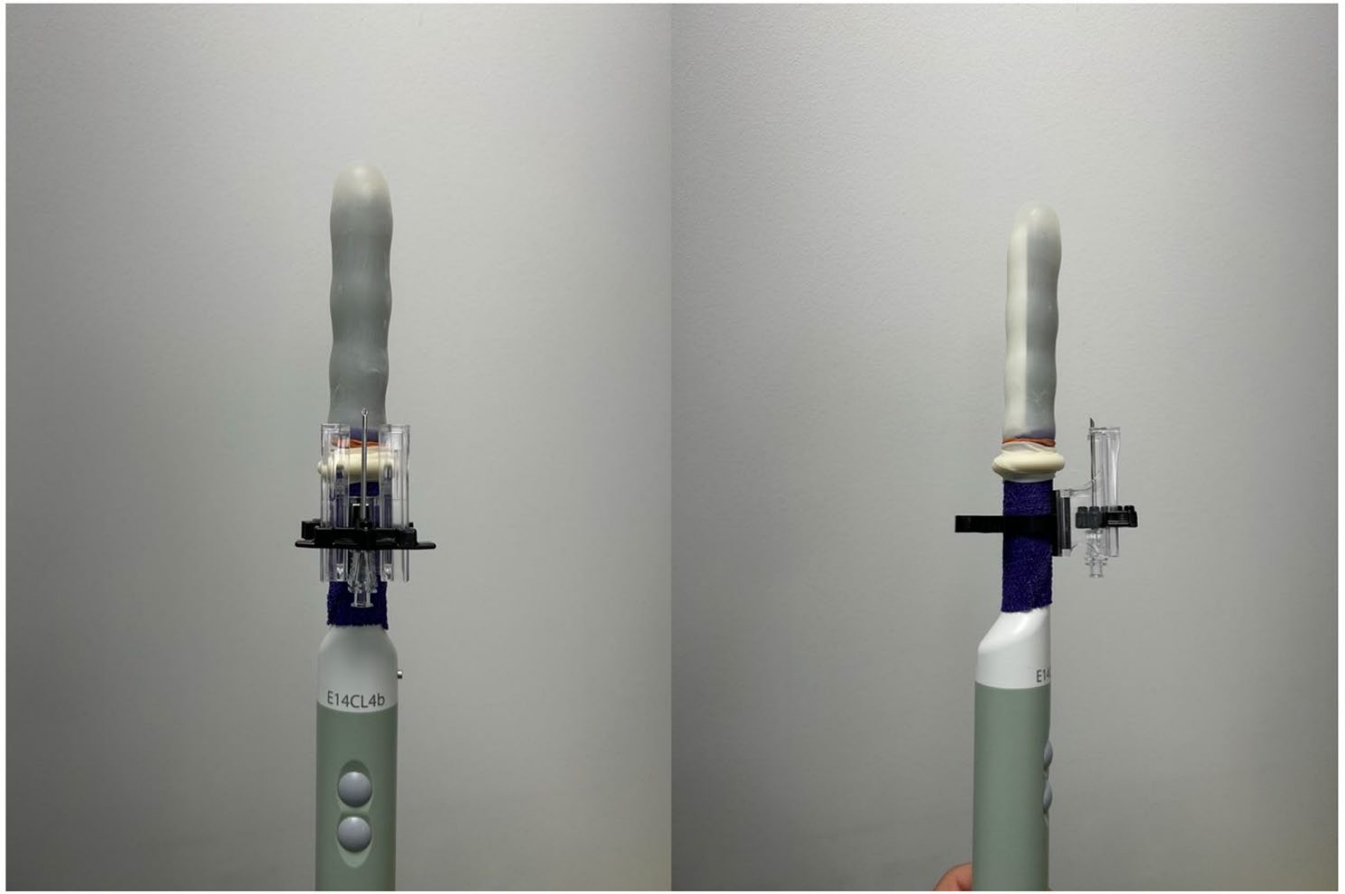
Biplane linear ultrasound probe 7 Hz with 3 M™ Coban™ wrapped around proximal shaft and a transducer cover with ultrasound gel secured with rubber bands at the base. PrecisionPoint access system over proximal shaft

A biplane linear ultrasound probe 7 Hz is used. 3 M™ Coban™ 2 layer compression system is wrapped around the proximal end of the ultrasound probe to increase the diameter and improve the grip for mounting the PrecisionPoint™ Transperineal access system. The transducer cover (Civco) tip is inverted. A 50 ml bladder syringe filled ultrasound gel is used to inject 5-10 ml of gel in the inverted tip being careful to evacuate any air bubbles that may be present in the gel surrounding the tip of the transducer probe. The cover is then fitted over the shaft of the probe and secured with two rubber bands at the base after wiping any excess gel from proximal end of the probe. (Fig. 3)

### Patient setup

2 × 11 ml local anaesthetic refrigerated gel syringes (we use gel that contains 2% Lidocaine) are inserted into the patient’s urethra. A penile clamp is applied over gauze around the distal shaft of the penis for 2 to 5 min. The patient is placed in the lithotomy position on the couch with a nurse or HCA at the head end to converse with the patient throughout the procedure.

### Procedure

The ultrasound probe with PrecisionPoint access system mounted is inserted into the lubricated rectum. The prostate is visualised, and the lateral aspect identified. The perineal skin is marked where the access needle is pointing. The perineal skin is sterilised with Chloraprep applicator. Ethyl chloride spray is applied to the perineal skin followed by subcutaneous infiltration of 5 ml 1% lidocaine with 1:200,000 adrenaline on each marked side. The access needle punctures the skin at the marked site and advanced into the perineal tissue. The spinal needle is advanced through the access needle. The neurovascular bundle at the most lateral aspect of the prostate lobe is identified between the prostate and the seminal vesicle, and infiltrated with 10 ml 1% lidocaine on each side, ensuring the injectate is in the correct plane. The ultrasound probe is removed from the rectum.

The penile clamp is removed, and patient is prepared with antiseptic solution, then draped. Two further 11 ml syringes of local anaesthetic and antiseptic gel are instilled into the urethra. The Rezūm™ device is primed to ensure irrigation is running appropriately and steam comes through the needle. The Rezūm™ device with cystoscope is advanced into the urethra and a urethrocystoscopy is performed. The configuration of the prostate is inspected, and a decision is made on the number of treatments to each lobe according to the length of the gland. The needle is deployed at each point of treatment within the prostate gland on either side.

Whilst rotating the delivery device 90 degrees laterally, the needle is deployed and fully inserted into the prostatic tissue up to the black depth marker ensuring that the emitter holes are within the prostatic tissue. The vapour activation button is pulled and held until the treatment cycle is complete. The vapour activation button is released, and the needle retraction button is pressed to retract the needle. Further treatments in the same manner are given as appropriate. The patient is monitored for pain throughout the procedure. The device is removed and a two way 14-16Fr urethral catheter is inserted urethrally with 10 ml water in the balloon. The drapes are removed, the patient taken to the recovery area for monitoring, and a Visual Analogue Pain score filled in, by the patient, to reflect the pain during the procedure itself (0 being no pain and 10 is maximum pain). We do not routinely give any further post operative analgesia either immediately or at discharge. The patient may take simple analgesia at home if they require it.

### Post-operative follow-up

The urethral catheter is removed in 5–7 days at the Urology out-patient clinic with post-void residual to ensure a successful removal. Patients are instructed to stop any alpha blockers at 6 weeks. Patients may contact the specialist nurse at any time postoperatively if there is a problem, however all patients are routinely followed-up at a dictated BPE clinic at approximately three months by the CNS with uroflowmetry study and residual volume. An IPSS [7] and IIEF-5 [8] qualitative questionnaire are also recorded at the time as this is considered as an ejaculatory-sparing procedure. The patients are also asked about any change to the quality of their ejaculate post-operatively.

### Data collection

We collect anonymised data retrospectively into a dedicated data collection tool to audit practice and ensure good quality patient care. As this was an audit, ethical approval was not required as per NHS Health research authority. We registered the audit with our local research and development department, NHS Fife. Data included patient demographics, size of prostate, PSA, comorbidities and medication history, pre-operative work up data in clinic, number of treatments on each lobe, post operative outcomes and complications. The intraoperative pain score is recorded immediately post operatively. The other parameters are recorded at the 3-month follow-up clinic appointment.

## Results

We have collected data on 13 Rezūm™ cases in the described way. Table 1 describes their demographics and outcome. The mean pain score during the procedure was 2.1 out of 10. No patients required additional analgesics or sedation during the procedure. Seven patients have had follow up in clinic so far. Regarding pre and post op changes, mean IPSS score decreased from 20.6 to 5.4, mean quality of life score from 4.6 to 1.3, mean maximum flow rate increased from 8.8 ml/s to 14.4 ml/s. There were no serious complications. All passed trial without catheter (1 patient failed first time and required a second attempt).

**Table 1:** Demographics and preliminary outcome from 13 daycase Rezūm™ cases under local anaesthetic without sedation

Median age (IQR)	66 (64–71)
**Pre-operative (13 patients)**	
Mean prostate size	61.2 cc
Mean PSA	2.3
Medications for LUTS	
*Alpha blocker*	8(61.5%)
*5 alpha reductase inhibitor*	7 (53.9%)
*Anticholinergic*	3 (23.1%)
*Phosphodiesterase 5 inhibitor*	0
*Mirabegron*	0
*Not on any medications*	2 (15.4%)
Pre-op catheter use	1 long term catheter for 1 year 1 intermittent self-catheter for 6 months
Mean Pre-op IPSS	20.6
Mean Pre-op QOL	4.6
Mean Pre-op Qmax	8.8 ml/s
Mean Pre-op Av flow	3.5 ml/s
Median PVR	63 (34–113)
**Operative (13 patients)**	
Number of treatments on each lobe	
*1*	6 patients
*2*	7 patients
Mean pain score (0–10)	2.1
**Post-operative (7 patients followed up so far)**	
Successful TWOC	13/13 (1 failed first TWOC)
Immediate Complications	- 1 UTI (treated with oral antibiotics)
Delayed complications	- 3 Bleeding (not requiring admission) - 3 retrograde ejaculation - 1 Erectile dysfunction
Mean Post-op IPSS	5.4
Mean Post-op QOL	1.3
Mean Post-op Qmax	14.4 ml/s
Mean Post-op Average flow	7.2 ml/s
Median PVR (IQR)	19 (1–40)

IQR = Interquartile range, LUTS = Lower urinary tract symptoms, IPSS = International prostate symptom score, QOL = Quality of life score, Qmax = Maximum flow rate, PVR = Post void residual.

## Discussion and conclusion

We have demonstrated the feasibility of delivering an out-patient local anaesthetic minimally invasive BOO procedure service. The mean patient pain score, 2.1/10, shows it is well tolerated, and the outcomes of improved IPSS (20.6 to 5.4) and QOL (4.6 to 1.3) are promising. These results are similar to both short term and long term outcomes described in the literature [1, 3] which describe a 40–50% improvement in IPSS and QOL.

The benefits of delivering Rezūm™ in a clinic setting as described, include reduced in-patient waiting list for BOO surgery, particularly in light of the increasing backlog in the post-COVID era and relocation of patients and resources from GA lists to the out-patient setting. From a patient perspective, the total duration of patient hospital stay is approximately 2 h, and fasting is not necessary. Patients avoid a general anaesthetic which is invasive, requires recovery time, and precludes the patient from driving or being alone after 24-hours. There is also a clear benefit and a treatment solution to frailer patients, or those with multiple or serious comorbidities for which the risks of a general anaesthetic are higher than the benefits of treatment. From our experience, patients are very satisfied with the service and prefer it to a GA day-case procedure, and preliminary outcomes look promising. Clearly the small patient numbers are a limitation to this study, but we aimed to demonstrate the feasible way in which this method can be delivered to aid urologists in setting up their own similar services. We are continuing to audit our practice and aim to publish further long-term outcomes on a larger cohort.

This service can be extrapolated to other minimally invasive surgical techniques such as UroLift^®^, iTIND etc., which we have started doing in our unit. We believe that this protocol is transferrable to other Urology units once the infrastructure and human resources is in place. There is a need for implementing a larger, well-designed study such as a randomised controlled trial to provide good evidence as to its non-inferiority, in order to improve the generalisability of this protocol.

## Data Availability

Data is provided within the manuscript.

## Abbreviations

BOO: Bladder outflow obstruction
BPE: Benign prostatic enlargement
CNS: Cancer nurse specialist
GA: General anaesthetic
HCA: Healthcare assistant
IIEF: International Index of Erectile Function
IPSS: International Prostate Symptom Score
IQR: Interquartile range
LA: Local anaesthetic
LUTS: Lower urinary tract symptoms
QOL: Quality of life score
